# Pigmented Villonodular Synovitis of the Knee Joint in a 10-Year-Old Patient Treated With an All-Arthroscopic Synovectomy: A Case Report

**DOI:** 10.7759/cureus.11929

**Published:** 2020-12-05

**Authors:** Frideriki Poutoglidou, Dimitrios Metaxiotis, Anastasios Mpeletsiotis

**Affiliations:** 1 Orthopaedic Department, Papageorgiou General Hospital of Thessaloniki, Thessaloniki, GRC

**Keywords:** arthroscopic synovectomy, pigmented villonodular synovitis, knee, paediatric

## Abstract

Pigmented villonodular synovitis (PVNS) is a relatively rare, benign lesion characterized by exuberant proliferation of the synovial tissue that most commonly affects the knee and hip joint. Magnetic resonance imaging (MRI) is the imaging modality of choice for the diagnosis of PVNS. The disease is confirmed histologically by examination of the synovial tissue removed. The mainstay of treatment is synovectomy, performed in an open, arthroscopic, or combined fashion. Although postoperative adjuvant external beam radiotherapy can improve the local recurrence rate, the course of the disease is not always uneventful. We present a rare case of a 10-year-old boy presented to our orthopaedic department with a four-month history of intermittent right knee pain and swelling. MRI revealed joint effusion and extensive nodular synovial proliferation suggestive of PVNS. An arthroscopic synovectomy was performed and histological examination confirmed the diagnosis. The postoperative course was uneventful. Clinical suspicion of PVNS is essential in children with chronic knee pain and swelling. Arthroscopic synovectomy is an effective and reliable treatment option.

## Introduction

Pigmented villonodular synovitis (PVNS) is a relatively rare, benign, proliferative disease that affects the synovial joints, bursas and tendon sheaths. The term “Pigmented Villonodular Synovitis” was established by Jaffe et al. in 1941 [1]. Granowitz and Mankin expanded the terminology by dividing PVNS into localized and diffuse forms [2].

The estimated incidence of PVNS ranges around 1.8 per million patients [3] and occurs equally in men and women. PVNS predominantly affects patients between 20 and 40 years old [4] and is rarely observed in the pediatric population [5,6]. The knee is mostly affected, in approximately 80% of the cases, followed by the hip, ankle, shoulder, and elbow [3,4].

Although there has been a great deal of speculation regarding the etiology of PVNS, it remains unknown. Previous reports suggest that PVNS is a result of chronic inflammation [7] or trauma-induced hemorrhage [8], but recent evidence in the literature supports its neoplastic origin [9]. Histologically, PVNS is characterized by mononuclear stromal cells, plump hyperplastic synovial cells and hemosiderin stained multinucleated giant cells [10], and resembles giant cell tumor of the tendon sheath and hemophilic synovitis. Clinical presentation is non-specific and includes pain, swelling, mechanical symptoms, limited range of motion and recurrent atraumatic hemarthrosis [11]. MRI provides an excellent delineation of the disease and is the most sensitive imaging study to evaluate PVNS [12].

The mainstay of treatment is surgical resection of the lesion in the localized form and total synovectomy in the diffusely involved joints, which can be performed with an open, arthroscopic, or a combined approach [13]. Radiation therapy and radiosynoviorthesis have been proposed to reduce recurrence rates [14].

In this study, we present a rare case of a 10-year-old boy with localized PVNS of the knee joint treated with an all-arthroscopic synovectomy.

## Case presentation

**Figure 1:**
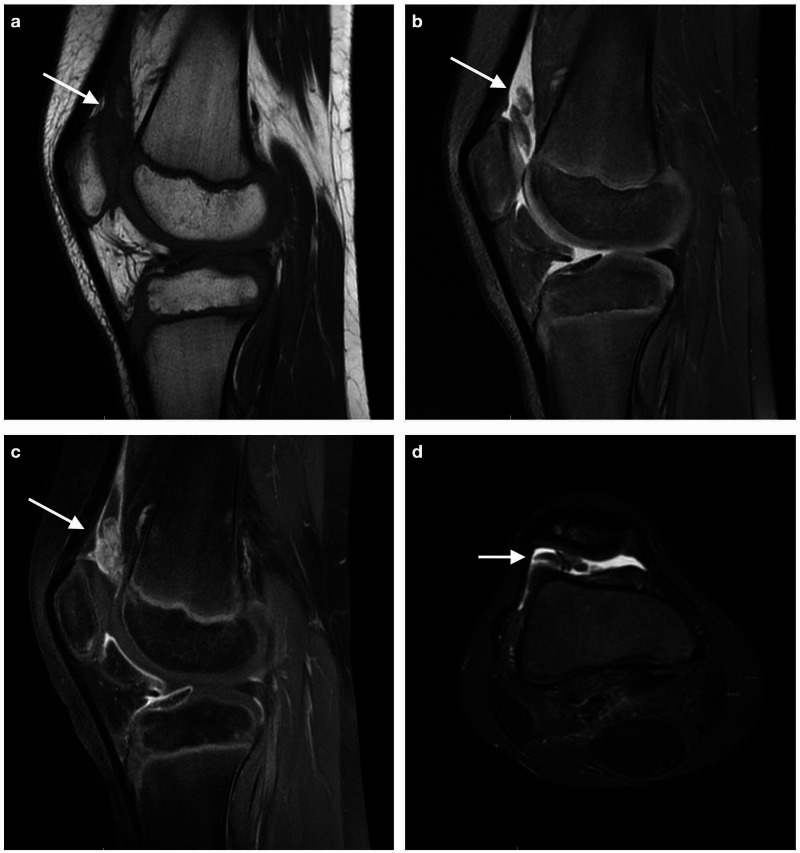
Magnetic Resonance Imaging of the right knee revealing extensive synovial proliferation, suggestive of pigmented villonodular synovitis (PVNS). Maximum amount of hypertrophic synovium in the suprapatellar pouch (arrow) a. Sagittal T2-weighted sequence, b. Sagittal PD fat suppressed sequence, c. Sagittal T1 FS sequence after administration of gadolinium (distinct enhancement), d. Axial STIR sequence

A 10-year-old boy with a four-month history of intermittent pain and swelling of his right knee was referred to our orthopaedic department for further evaluation. Despite prolonged rest, joint immobilization and nonsteroidal anti-inflammatory treatment, the patient complained of pain that worsened with activity and stiffness. There were no associated symptoms or other joint involvement and no history of trauma. Physical examination revealed joint effusion, palpable synovial thickening, and moderate restriction of knee range of motion. There were no signs of inflammation or ligament laxity. Radiographic findings of the right knee were unremarkable. Laboratory workup included a complete blood count (CBC), a basic metabolic panel, and serological markers for rheumatoid diseases and they were all within the normal range. An MRI of the right knee revealed joint effusion and extensive nodular synovial proliferation suggestive of PVNS (Figure 1).

**Figure 2:**
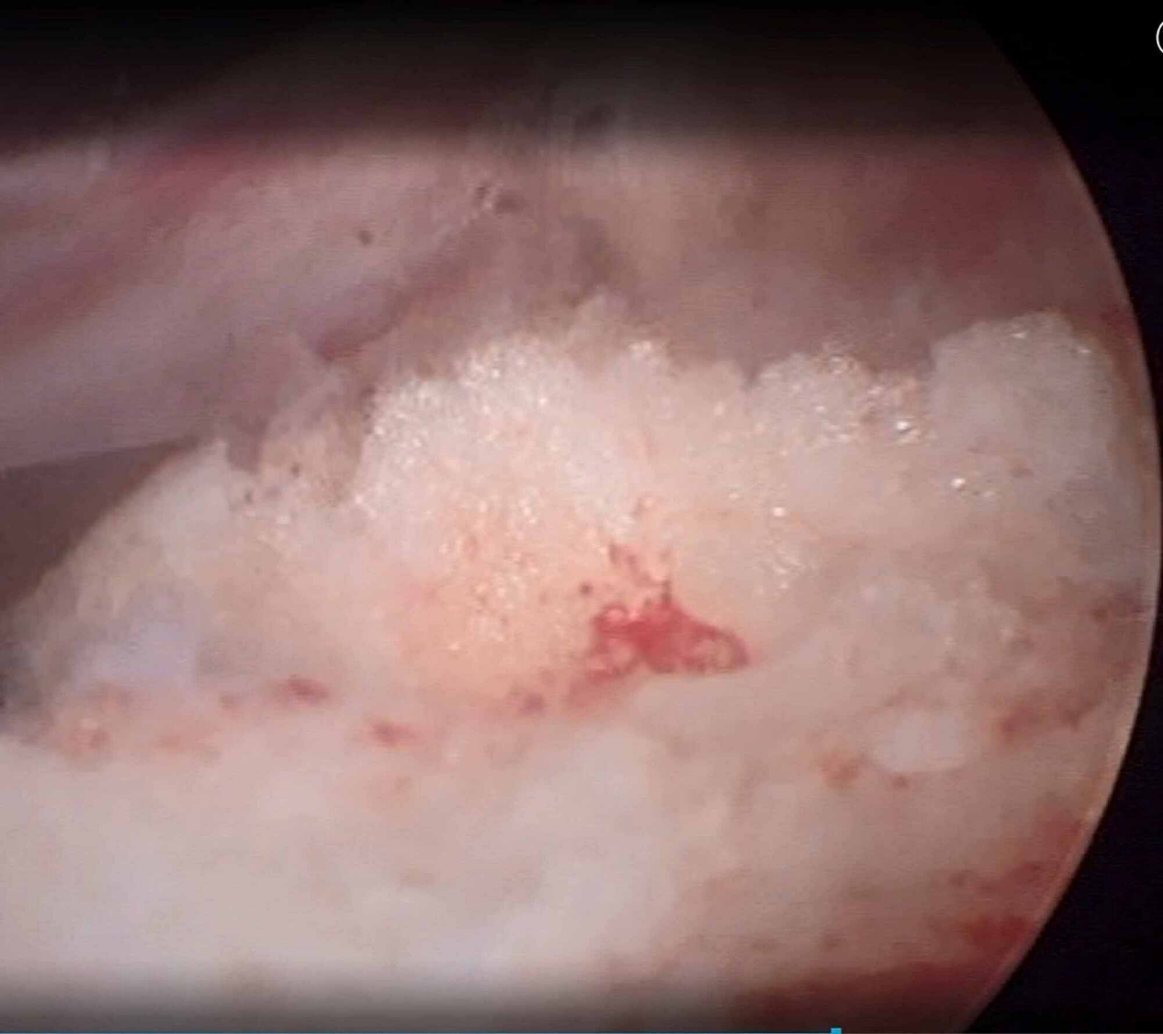
Arthroscopic view of pigmented villonodular synovitis (PVNS) lesion located at the suprapatellar pouch

**Figure 3:**
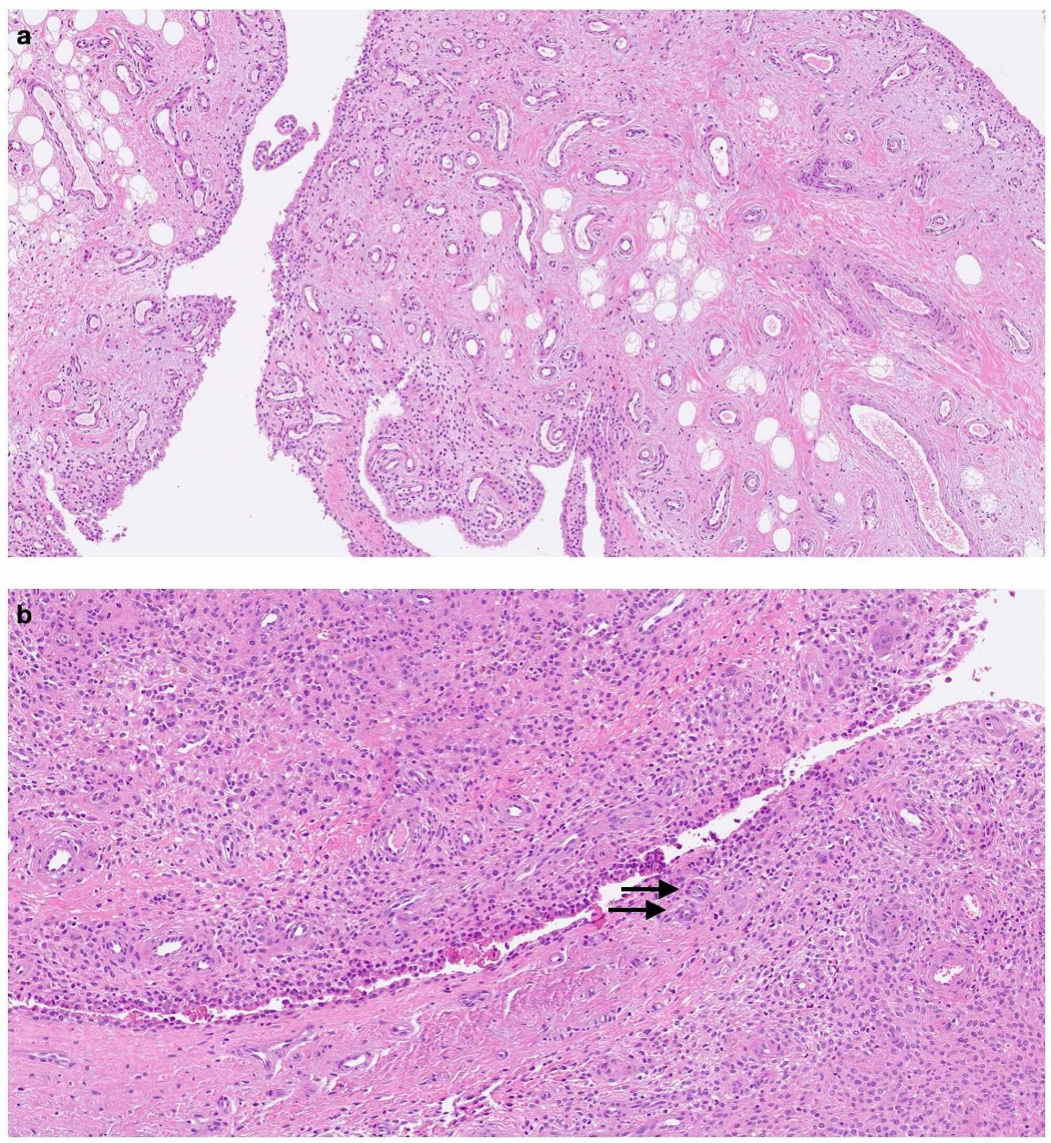
Photomicrographs of H&E stain samples of the resected synovium consistent with pigmented villonodular synovitis (PVNS). a. Villous and nodular configuration, b. Synovium hypertrophy, scattered multinucleated giant cells (arrows) H&E: hematoxylin and eosin stain

The patient was taken to the operating room for an arthroscopic synovectomy (Figure 2). Under general anesthesia, the patient was placed in a supine position and the procedure was performed through an all-arthroscopic fashion using the standard anteromedial and anterolateral portals. Suprapatellar pouch had the maximum amount of hypertrophic synovium. Hemostasis was achieved with electrocautery and a compression bandage was applied to prevent postoperative hemarthrosis. The hypertrophic synovium was sent for histopathologic examination. Histology showed vascular villi with hyperplastic synovial cells and hemosiderin stained multinucleated giant cells (Figure 3) that confirmed the diagnosis of PVNS.

The postoperative clinical course was uneventful. Physical therapy began on postoperative day two, once the drain was removed. Suture removal was done on day 14. At the two-month follow-up, the patient was asymptomatic, had a full range of motion and there was no evidence of recurrence. One year after the initial diagnosis, the patient remained symptom-free.

## Discussion

PVNS is a relatively rare disease with non-specific symptoms and insidious onset that often lead to a delayed diagnosis. Schwartz et al. suggest a five-year average duration of symptoms before the patient seeks medical attention [15]. The vague clinical presentation combined with the fact that PVNS mainly affects patients between their third and fourth decade of life [4] makes the diagnosis in the pediatric population quite challenging.

Various imaging modalities are often necessary to confirm the diagnosis. Radiographs may show cystic erosions, however, in the early stages of the disease are usually negative [16]. MRI is the only available imaging modality that is able to clearly depict the hypertrophic synovium and any extra-articular extension. Typically, PVNS has a low to isointense signal on both T1 and T2 weighted sequences due to the hemosiderin deposits. “Blooming artifact”, seen on T2 weighted gradient-echo sequences, is a result of magnetic susceptibility of the iron in hemosiderin [12].

Treatment of PVNS remains challenging. The aim of PVNS management is the removal of all the abnormal synovial tissue to relieve pain and avoid recurrence. Total synovectomy, that can be performed in an arthroscopic, open or combined fashion, is the mainstay of treatment [13]. Previous reports support similar results with open and arthroscopic synovectomy [17]. Arthroscopy is a minimally invasive procedure that offers a faster return to normal activity. In our patient, we chose an arthroscopic approach in an effort to minimize postoperative pain and reduce recovery time.

Local recurrence is the most frequent complication, particularly in the diffuse type of PVNS. Verspoor et al., in a large series of 107 patients, report an overall recurrence rate of 72% and 22% for the diffuse and the localized PVNS subtypes, respectively [18]. External beam radiation, alone or as adjuvant therapy after synovectomy, has been proposed to reduce recurrence rates. However, radiation is not a complication-free therapy. Stiffness, skin necrosis and radiation-induced malignancies have been reported [19]. Recently, new targeted therapies have been developed for the treatment of PVNS. Recent research has revealed that imatinib, a tyrosine kinase inhibitor, has a critical role in controlling the disease and relieving the symptoms, making it a promising therapeutic agent [20]. Nevertheless, potential toxicities have been reported and the cost of treatment is considerably high.

## Conclusions

Although PVNS is a relatively rare disease, particularly in the pediatric population, it should be considered in the differential diagnosis of chronic joint swelling and pain. MRI remains the imaging modality of choice for the diagnosis of PVNS. Arthroscopic synovectomy, when performed by an experienced surgeon, not only guarantees a faster recovery but also can achieves satisfactory control of the disease and low recurrence rates.
